# Human infection with a novel reassortant Eurasian-avian lineage swine H1N1 virus in northern China

**DOI:** 10.1080/22221751.2019.1679611

**Published:** 2019-10-29

**Authors:** Xiaoyan Li, Liru Guo, Caixia Liu, Yanhui Cheng, Mei Kong, Lei Yang, Zhichao Zhuang, Jia Liu, Ming Zou, Xiaochun Dong, Xu Su, Qing Gu

**Affiliations:** a Tianjin Centers for Disease Control and Prevention, Tianjin, People’s Republic of China; b Jizhou District Center for Disease Control and Prevention, Tianjin, People’s Republic of China; c Chinese National Influenza Center, National Institute for Viral Disease Control and Prevention, Chinese Center for Disease Control and Prevention, Beijing, People’s Republic of China

**Keywords:** Influenza A virus, EAS-H1N1, triple-reassortant, molecular characteristics, Phylogenetic analysis

## Abstract

Influenza A virus infections occur in different species, causing mild to severe respiratory symptoms that lead to a heavy disease burden. Eurasian avian-like swine influenza A(H1N1) viruses (EAS-H1N1) are predominant in pigs and occasionally infect humans. An influenza A(H1N1) virus was isolated from a boy who was suffering from fever and headache and designated as A/Tianjin-baodi/1606/2018(H1N1). Full-genome sequencing and phylogenetic analysis revealed that A/Tianjin-baodi/1606/2018(H1N1) is a novel reassortant EAS-H1N1 containing gene segments from EAS-H1N1 (HA and NA), classical swine H1N1(NS) and A(H1N1)pdm09(PB2, PB2, PA, NP and M) viruses. The isolation and analysis of A/Tianjin-baodi/1606/2018(H1) provide further evidence that EAS-H1N1 poses a threat to human health and greater attention should be paid to surveillance of influenza virus infection in pigs and humans.

## Introduction

Influenza A virus can be classified into different subtypes based on antigenic variation in two surface glycoproteins, haemagglutinin (HA) and neuraminidase (NA) [1]. Genetic mutations that encode a range of amino acid substitutions result in antigenic changes in the surface glycoproteins and lead to immunologic escape, which is known as antigenic drift [2–4]. In addition, gene segment exchange between two or more viruses, known as genetic reassortment, can also play an important role in the evolution of new influenza viruses [5–6].

Influenza A virus infection occurs in different animals, including pigs. Both *α*-2,3-linked sialic acids (Sias) (avian influenza virus receptor) and *α*-2,6-linked Sias (human influenza virus receptor) are found in pigs; hence, they are recognized as genetic mixing vessels for humans and avian influenza viruses [7–8]. Classical H1N1 swine influenza viruses (SIVs) cause sporadic zoonotic infections and a reassortant, swine-origin influenza A H1N1 virus (A(H1N1)pdm09) caused an influenza pandemic among humans in 2009 [9].

The first EAS-H1N1 human infection was reported in Switzerland in 1986 [10]. Since then, several human infections with EAS-H1N1 have occurred in European countries [11–12]. China is the largest pork-producing country in the world and it is known that EAS-H1N1 circulates in swine [13–17]. Furthermore, four zoonotic cases related to EAS-H1N1 infection have been reported in China, indicating the potential to cause a human influenza pandemic [18–21]. In this study, we isolated and characterized a novel triple-reassortant EAS-H1N1 from a nine-year-old boy.

## Materials and methods

### Epidemiologic information, sample collection and virus identification

A nine-year-old boy, who lived in the countryside with his family, presented with fever of 38.7°C and started coughing with pharyngeal pain and headache on 10 December 2018. The following day, the boy was sent directly to Baodi People’s Hospital (an influenza surveillance network hospital) and a throat-swab specimen was collected and transferred to Jizhou District Center for Disease Control and Prevention (Jizhou CDC). A second throat-swab sample was collected on 14 December. Throat swabs from all his close contacts and 28 environmental specimens (including cage surface wipes, poultry and swine excrement and drinking water) were collected for influenza virus detection using real-time reverse transcription polymerase chain reaction (real-time RT–PCR). Nucleic acid was extracted from 500 µl of the clinical specimen using NucliSENS easyMAG (BioMetrix, France) and real-time RT–PCR was carried out using FluA real-time PCR kit (Bojie, Shanghai, China) in accordance with the manufacturer’s instructions.

### Virus isolation and antigenic characteristics

Clinical specimen was inoculated onto Madin–Darby Canine Kidney cells (MDCK) and cultured with serum-free minimum essential medium (MEM; Gibcol, USA) in the presence of 2.0 µg/ml of trypsin (Sigma, USA) at 34°C [22]. Checking for cytopathic effect (CPE) was conducted every day. Medium was collected when the CPE was up to 75–100% cells. Virus haemagglutination titre (HAT) and haemagglutination inhibition (HI) titres using antisera raised against specific A(H1N1)pdm09 reference viruses, distributed by the Chinese National Influenza Center (CNIC), were determined with 1.5% human-type “O” erythrocytes. If the HI titre of test viruses with the reference antiserum was less than eight-fold different to that of the reference virus, this result meant the test viruses were antigenically similar.Figure 1.Phylogenetic analysis of eight gene segments on A/Tianjin-baodi/1606/2018(H1). The reliability of the trees was assessed via bootstrap analysis with 1000 replications. *Pandemic H1N1* means *A(H1N1)pdm09*.

**Figure:**
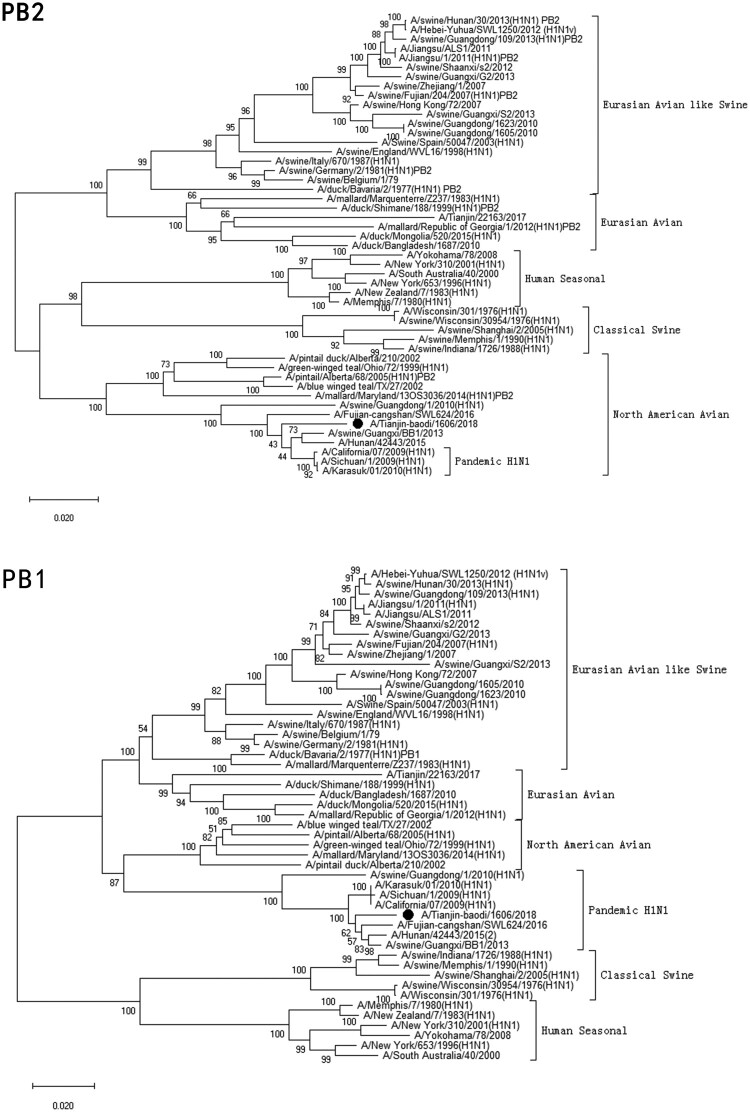

**Figure:**
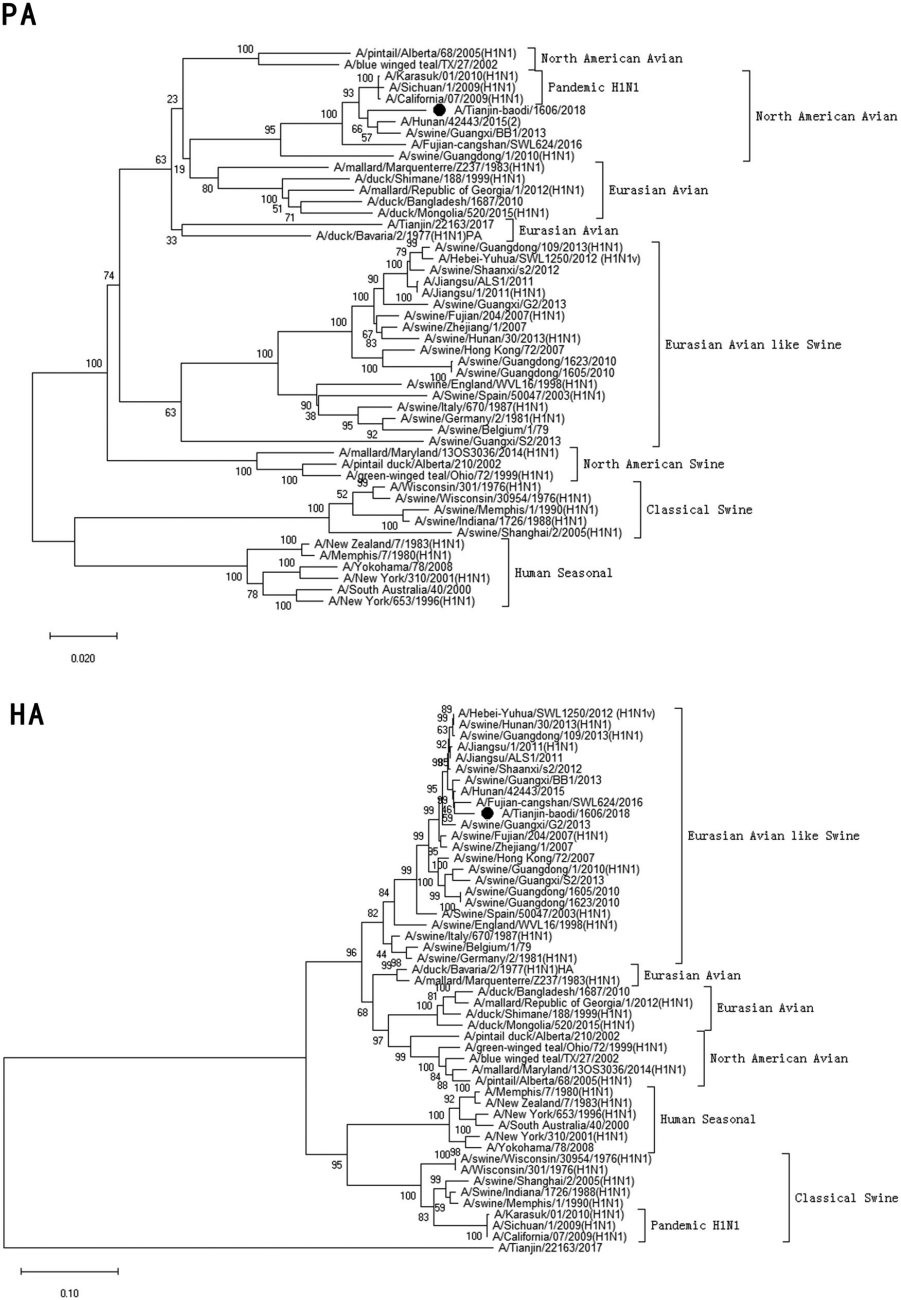

**Figure:**
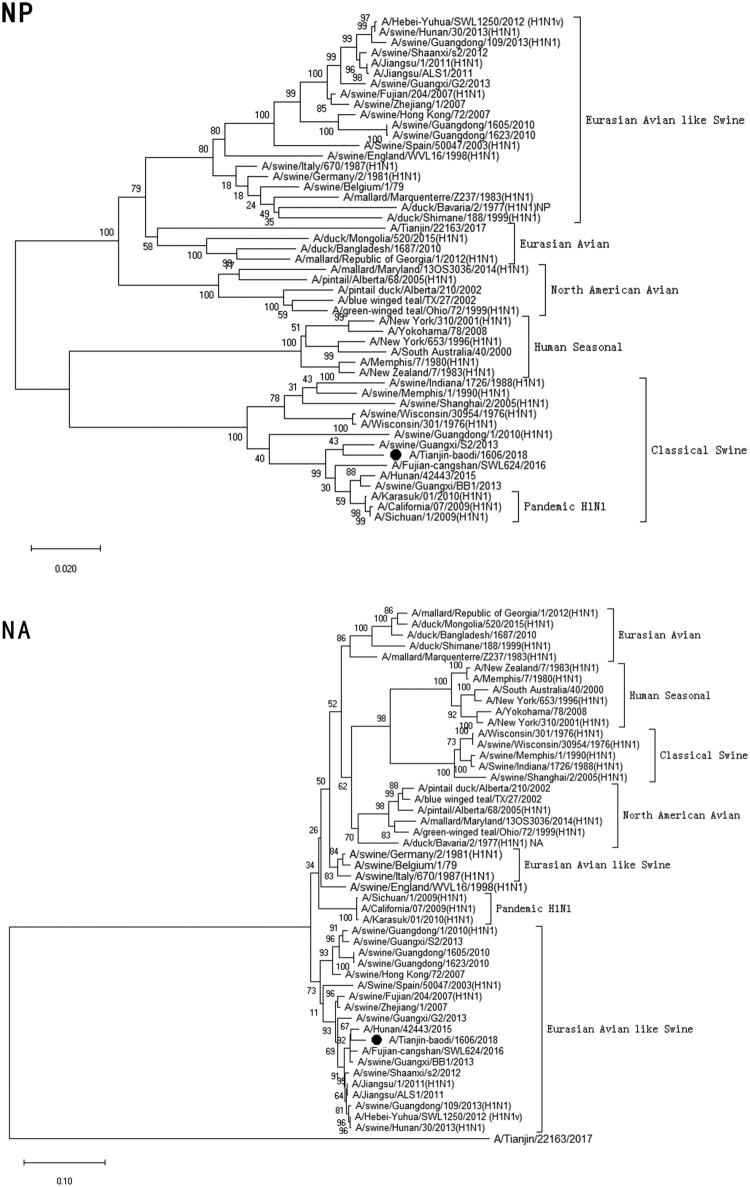

**Figure:**
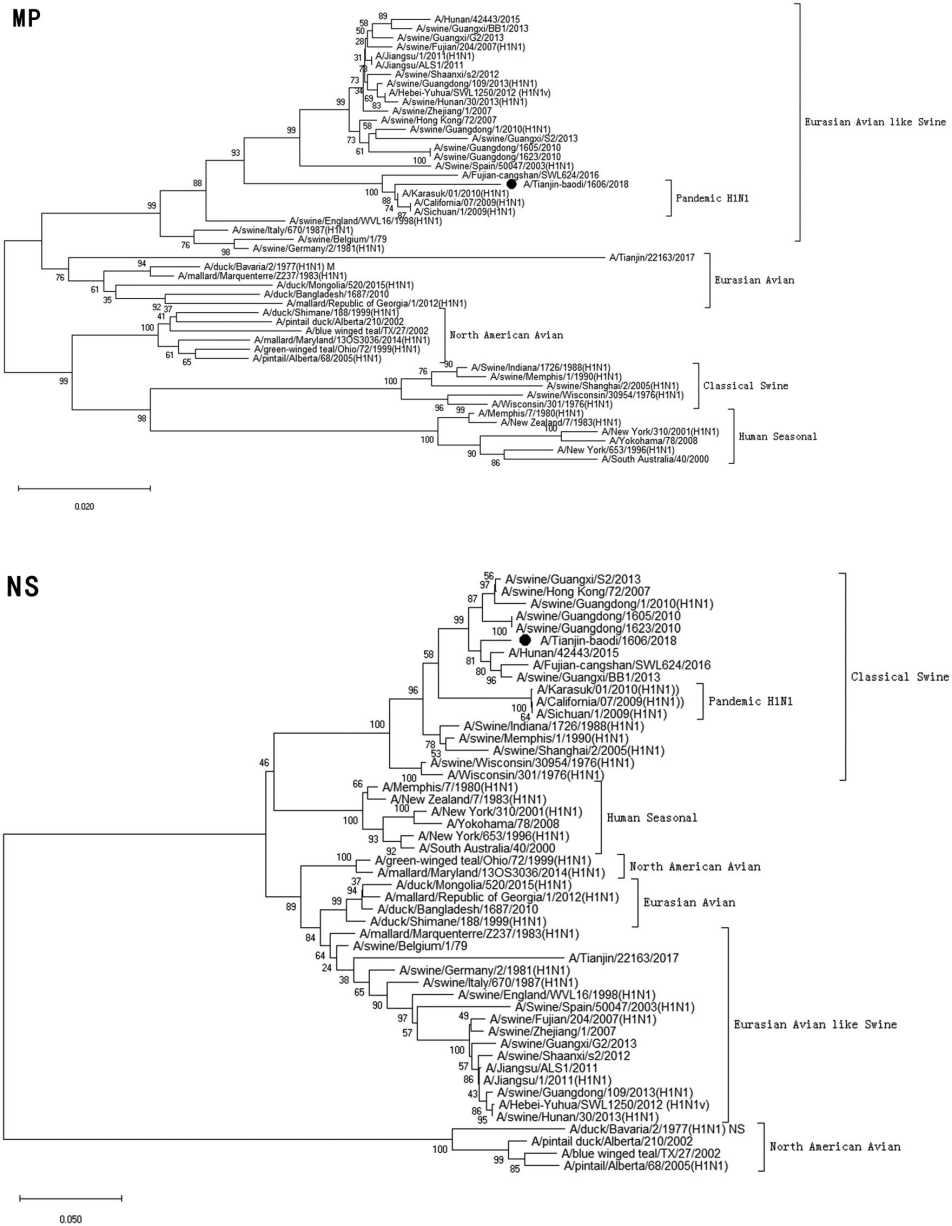

### Antiviral susceptibility

The susceptibility of A/Tianjin-baodi/1606/2018(H1N1) to the NA inhibitor oseltamivir carboxylate (Roche Diagnostics GmbH) was evaluated using an NA-Fluor Influenza Neuraminidase Assay Kit (Applied Biosystems). Oseltamivir-resistant virus (A/Texas/23/2012(H1)Y275) and oseltamivir-sensitive virus (A/Texas/23/2012(H1)H275), provided by CNIC, were used as controls. The half maximal inhibitory concentration (IC_50_), which represented the concentration of oseltamivir that could inhibit 50% of NA activity, was used to evaluate the antiviral susceptibility of the virus. Viruses are considered to show normal inhibition (NI) if IC_50_ is increased no more than 10-fold compared to the oseltamivir-sensitive control virus, reduced inhibition (RI) if the IC_50_ is increased 10–100-fold and highly reduced inhibition (HRI) if IC_50_ is increased >100-fold (see the WHO web site https://www.who.int/influenza/gisrs_laboratory/antiviral_susceptibility/nai_overview/en/).

### Genome sequencing and phylogenetic analysis

Viral RNA was extracted from 140 μl of virus stock using an RNeasy Mini Kit (QiaGen, Germany) as described by the manufacturer. Primers specific for each gene segment of influenza A virus were used for RT–PCR [23] and the products were purified using a QIAamp Gel Extraction Kit (QiaGen, Germany). Whole genome sequencing was carried out using an Illumina MiniSeq platform (Illumina, USA). Sequences were edited using the Lasergene sequence analysis software package (DNAStar, Madison, WI, USA). Sequence alignments and phylogenetic analysis were performed with MEGA X software and neighbour-joining trees were assembled with bootstrap values determined from 1000 replicates. All reference sequences were downloaded from the EpiFlu database of the Global Initiative on Sharing All Influenza Data (GISAID). The key molecular features were observed and analysed through the alignment with other reference viruses.

## Results

### The patient and epidemiology survey

A previously healthy nine-year-old boy, with no history of travel, presented with influenza-like illness (ILI) symptoms on 10 December 2018, having a fever of 38.7°C, pharyngeal pain and headache. He recovered within a week with neither hospitalization nor oseltamivir treatment. A retrospective investigation was conducted to identify the potential source of infection and any other possible cases. The patient had no contact with individuals showing ILI symptoms within 10 days before onset of his symptoms and his family raised eight chickens in captivity while their neighbours raised pigs in captivity. He lived with his parents and grandparents. None of his close contacts developed ILI symptoms during the period of the investigation.

### Sample identification and viral isolation

The first throat-swab specimen was sent to the Jizhou CDC influenza surveillance network laboratory. By real-time RT–PCR, the sample was positive for influenza A virus, but negative for H3N2, H1N1pdm09, H5, H7 and H9 influenza viruses. The second specimen was negative for influenza virus. The influenza-positive specimen was subsequently transferred to Tianjin CDC for further investigation. Influenza A-positivity was confirmed and shown to be caused by an H1N1 virus which was different from H1N1pdm09. The virus, designated as A/Tianjin-baodi/1606/2018(H1N1) (TJ/1606/18), was isolated using MDCK cells. All specimens taken from his close contacts and the environment (including cage surface wipe swabs, poultry and swine excrement and drinking water of the animals) were negative for influenza virus.

### Antigenic characteristics

Results from HI revealed that TJ/1606/18 was antigenically similar to A(H1N1)pdm09 virus vaccine strains A/California/07/2009(H1N1) and A/Michigan/45/2015(H1N1) (Table 1).

**Table 1.** Antigenic analysis of TJ/1606/18 using HI assay.

Virus	HI antibody titres of ferret antiserum against^a^		
	A/California/7/2009C	A/Michigan/45/2015E	A/Michigan/45/2015C
A/California/7/2009(H1N1)C^b^	**2560**	160	640
A/Michigan/45/2015(H1N1)E^c^	1280	**1280**	2560
A/Michigan/45/2015(H1N1)C^b^	2560	2560	**2560**
A/Tianjin-baodi/1606/2018(H1N1)	640	640	1280

^a^Homologous titres are shown in boldface. Antiserum was obtained from ferret after immunized with virus stock once.

^b^Viruses were isolated using MDCK cells.

^c^Viruses were isolated using embryonated hens’ eggs.

### Antiviral susceptibility

TJ/1606/18 displayed an NI phenotype, 1.2-fold increase in IC_50_ compared to the oseltamivir-sensitive control virus, with the NA inhibitor oseltamivir (Table 2).

**Table 2.** Antiviral susceptibility.

Virus^a^	IC50 (95% confidence interval)
A/Tianjin-baodi/1606/2018(H1N1)	0.46 (0.30–0.62)
A/Texas/23/2012(H1)H275 (NAI sensitive)	0.37 (0.27–0.47)
A/Texas/23/2012(H1)Y275 (NAI resistance)	55.50 (45.70–65.31)

^a^NAI: neuraminidase inhibitor.

### Molecular characteristics

Full-length sequences of the 8 gene segments of TJ/1606/18 (PB2, PB1, PA, HA, NP, NA, M and NS) were obtained, consisting of 2280, 2274, 2151, 1701, 1515, 1410, 982 and 838 nucleotides (only the nucleotides in the Open Reading Frame were calculated), respectively. Sequences of all eight segments have been submitted to GISAID, accession numbers: EPI1431720-EPI1431727.

Phylogenetic analysis revealed that TJ/1606/18 was a novel EAS-H1N1 containing genes from Eurasian avian-like swine H1N1 (HA and NA), A(H1N1)pdm09 (PB2, PB1, PA, NP and M), and classical swine H1N1 (NS) (Figure 1 and Table 3). All eight segments shared 93.6–97.9% and 94.9–97.1% nucleotide identity with A/Hunan/42443/2015 and A/Fujian-cangshan/SWL624/2016, respectively (Table 4), with94.5–98.8% and 94.0–99.6% identities in deduced amino acids (Table 5). However, the gene segments PA, NP and M of TJ/1606/18 showed the highest nucleotide homologies, 97%, 97.1%, and 98.1% respectively, with A/California/07/2009 virus (Table 4) and corresponding amino acid homologies of 98.5%, 98.6% and 99–100% (Table 5).

**Table 3.** Genetic origin of A/TJ/1606/18 based on phylogenetic analyses.

	Lineage assigned to gene segment							
Isolates	PB2	PB1	PA	HA	NP	NA	M	NS
A/California/07/2009(H1N1)	PDM	PDM	PDM	PDM	PDM	PDM	PDM	PDM
A/Jiangsu/1/2011(H1N1)^a^	EAS	EAS	EAS	EAS	EAS	EAS	EAS	EAS
A/Fujian-cangshan/SWL624/2016(H1N1)^a^	PDM	PDM	PDM	EAS	PDM	EAS	PDM	CS
A/Hebei-yunhua/SWL1250/2012(H1N1)^a^	EAS	EAS	EAS	EAS	EAS	EAS	EAS	EAS
A/Hunan/42443/2015(H1N1)^a^	PDM	PDM	PDM	EAS	PDM	EAS	EAS	CS
A/Tianjin/22163/2017(H7N9)^b^	EAS	EAS	EAS	AIV	EAS	AIV	EAS	EAS
A/swine/Guangdong/1/2010(H1N1)	PDM	PDM	PDM	EAS	PDM	EAS	EAS	CS
A/swine/Tianjin/9/2013(H1N1)^c^	PDM	PDM	PDM	EAS	PDM	EAS	EAS	CS
**A/Tianjin-baodi/1606/2018(H1N1)**	**PDM**	**PDM**	**PDM**	**EAS**	**PDM**	**EAS**	**PDM**	**CS**

Notes: PDM, genes closest homology to A(H1N1)pdm09 viruses; EAS, genes with closest homology to Eurasianavian-like swine influenza viruses; CS, genes with closest homology to classical swine influenza viruses; AIV, avian influenza viruses.

^a^Human infection with Eurasian avian-like swine influenza virus.

^b^Human infection with avian influenza virus H7N9 in Tianjin.

^c^Noveltriple-reassortant H1N1 swine influenza viruses in pigs in Tianjin, China [35].

**Table 4.** Nucleotide homology analysis of the eight gene segments of A/TJ/1606/18.

Isolates	A/Tianjin-baodi/1606/2018(H1) (nucleotide identities %)							
	PB2	PB1	PA	HA	NP	NA	M	NS
A/California/07/2009(H1N1)	96.6	97.4	97.0	66.6	97.1	88.2	98.1	91.2
A/Jiangsu/1/2011(H1N1)^a^	80.6	81.2	81.2	96.6	79.8	96.9	94.1	79.6
A/Fujian-cangshan/SWL624/2016(H1N1)^a^	95.2	97.1	94.9	96.1	95.6	97.1	97.1	96.4
A/Hebei-yunhua/SWL1250/2012(H1N1)^a^	80.1	81.5	80.8	96.4	80.0	96.7	93.6	78.6
A/Hunan/42443/2015(H1N1)^a^	97.0	97.9	96.9	97.5	96.4	97.2	93.6	97.0
A/Tianjin/22163/2017(H7N9)^b^	81.2	81.8	86.2	24.3	79.8	29.9	84.4	75.6
A/swine/Guangdong/1/2010(H1N1)	92.7	92.8	92.0	91.7	92.3	85.1	93.4	94.7

^a^Human infection with Eurasian avian-like swine influenza virus.

^b^Human infection with avian influenza virus H7N9 in Tianjin.

**Table 5.** Amino acid homology analysis of A/TJ/1606/18 proteins.

Isolates	A/Tianjin-baodi/1606/2018(H1) (identities %)										
	PB2	PB1	PB1-F2	PA	HA	NP	NA	M1	M2	NS1	NS2
A/California/07/2009(H1N1)	98.3	98.9	98.0	98.5	78.9	98.6	92.1	100	99.0	88.6	89.0
A/Jiangsu/1/2011(H1N1)^a^	93.8	94.5	64.0	92.3	97.0	92.2	96.4	98.8	93.8	78.5	87.7
A/Fujian-cangshan/SWL624/2016(H1N1)^a^	97.2	98.7	94.0	97.1	97.2	98.4	97.3	99.6	97.9	94.7	94.5
A/Hebei-yunhua/SWL1250/2012(H1N1)^a^	93.7	94.3	62.0	92.5	97.4	92.0	96.0	98.8	93.8	77.2	84.9
A/Hunan/42443/2015(H1N1)^a^	98.0	98.8	98.0	98.7	97.7	98.0	97.9	98.4	94.8	95.2	94.5
A/Tianjin/22163/2017(H7N9)^b^	96.3	95.5	56.0	95.0	42.4	92.6	48.9	92.1	88.7	75.4	83.6
A/swine/Guangdong/1/2010(H1N1)	96.4	97.4	90.0	95.5	92.4	97.4	93.6	99.6	93.8	92.1	89.0

^a^Human infection with Eurasian avian-like swine influenza virus.

^b^Human infection with avian influenza virus H7N9 in Tianjin.

The key molecular features of TJ/1606/18 known to be associated with increased virulence in mammals, mammalian transmissibility and antiviral susceptibility were shown in Table 6. TJ/1606/18 contained the amino acid motif PSIQSR↓GL at the HA1/HA2 cleavage site, a characteristic of influenza viruses with low pathogenicity [24]. Furthermore, seven potential glycosylation sites (N-X-S/T) were found at positions 27, 28, 40, 212, 291, 498 and 557 in the HA protein of the isolated virus. TJ/1606/18 had 190D and 225E in HA, indicative of increased binding to swine or human receptors.

**Table 6.** Molecular analysis of A/TJ/1606/18 compared to other viruses.

Gene product	Function	Amino acid substitution	Virus^a^						
			TJ	FJ	HN	HB	JS	GD	CA
HA	Altered receptor specificity	E190D	**D**	**D**	**D**	**D**	**D**	V	**D**
		D225E	**E**	**E**	**E**	**E**	**E**	**E**	D
NA	Antiviral resistance (oseltamivir)	H275Y	H	H	H	H	H	H	H
		N295S	N	N	N	N	N	N	N
PB2	Enhanced polymerase activity	L89V	**V**	**V**	**V**	**V**	**V**	**V**	**V**
	Virus replication in mammals	Q591R	**R**	Q	**R**	Q	Q	**R**	**R**
		E627K	E	E	E	E	E	E	E
		D701N	D	D	D	N	N	D	D
PB1	Between species transmission	X99H	**H**	**H**	**H**	**H**	**H**	**H**	**H**
		I368V	I	I	I	I	I	I	I
PA	Increased polymerase activity in mice	L336M	**M**	**M**	**M**	L	L	L	**M**
	Species-associated signature positions	K356R	**R**	**R**	**R**	K	K	K	**R**
		S409N	**N**	**N**	**N**	**N**	**N**	**N**	**N**
M1	Increased virulence in mice	T215A	**A**	**A**	**A**	**A**	**A**	**A**	**A**
M2	Antiviral resistance (amantadine)	S31N	**N**	**N**	**N**	**N**	**N**	**N**	**N**
NS1	Increased virulence in mice	P42S	**S**	**S**	**S**	**S**	**S**	**S**	**S**
NP	Mammalian-adaptive and increased virulence in mice	Q357K	**K**	**K**	**K**	Q	Q	**K**	**K**

^a^TJ, A/Tianjin-baodi/1606/2018(H1N1);FJ, A/Fujian-cangshan/SWL624/2016(H1N1); HN, A/Hunan/42443/2015(H1N1);HB, A/Hebei-yunhua/SWL1250/2012(H1N1);JS, A/Jiangsu/1/2011(H1N1); GD, A/swine/Guangdong/1/2010(H1N1);CA, A/California/07/2009(H1N1)pdm09. For each virus, amino acid positions carrying the substitution are highlighted.

The amino acid substitutions (H275Y and N295S) associated with reduced susceptibility to NA inhibitors were not observed in TJ/1606/18 NA, suggesting that the isolated virus was sensitive to antiviral drugs oseltamivir and zanamivir. This was consistent with the results of antiviral susceptibility test. However, the M2 protein had S37N amino acid substitution like A(H1N1)pdm09 viruses, indicative of resistance to the antiviral drugs amantadine and rimantadine [25–26].

In PB1 polymerase, TJ/1606/18 owned 99H and 368I. The TJ/1606/18 PB1-F2 protein is unlikely to function as the reading frame was interrupted by stop codons at positions equivalent to amino acid residues 12 and 86.

In addition, several amino acid substitutions related to virus virulence or host adaption have been reported, including L89V, Q591R, E627K, and D701N in PB2 polymerase, L336M, K256R and S409N in PA, T215A in M1 protein, P42S in NS1and Q357K in NP protein [27–33]. TJ/1606/18gene sequences encoded all but two, E627K and D701N in PB2 polymerase, of these amino acid substitutions (Table 6).

## Discussion

Influenza virus infection usually causes substantial mortality and morbidity. Pigs play an important role in the generation of novel influenza viruses with pandemic potential because they may be infected with both humans and avian influenza virus [34]. Currently, influenza viruses of subtypes H1N1, H3N2 and H1N2 are known to co-circulate in pigs [14]. Since 2005, the EAS-H1N1 viruses have become dominant [13–14] after their long-term evolution, the EAS-H1N1 viruses in China have been reported to preferentially bind to human-type receptors, and some of the viruses tested were transmitted in ferrets by respiratory droplets. This suggests that among the influenza viruses currently circulating in animals, the EAS-H1N1 SIVs pose the greatest pandemic threat [13].

Sun et al. reported that novel triple-reassortant EAS-H1N1 SIVs, containing gene segments from A(H1N1)pdm09 (PB1, PB2, PA and NP), EAS-H1N1 (HA, NA and M) and Classical SIVs (NS),were isolated from pigs in Tianjin, together with human-like H1N1, classical swine H1N1 and Eurasian swine H1N1 viruses [35], which indicated that multiple genetic lineages of swine H1N1 viruses were co-circulating in the swine population in Tianjin, China. Here, we show that TJ/1606/18 had close antigenic and genetic relationships with EAS-H1N1 viruses that were circulating in pigs in China. As the gene sequences can’t be available from GenBank or GISAID, we couldn’t analyse the detailed molecular characteristics between TJ/1606/18 and the novel triple-reassortant EAS-H1N1 isolated in pigs in Tianjin (mentioned above), and based on the phylogenetic trees published by Sun et al. in 2013 [35], it can be recognized that the novel triple-reassortant EAS-H1N1 viruses were closely related to TJ/1606/18, with only a difference that M gene derived from EAS-H1N1. This means EAS-H1N1 viruses have continued to circulate in pigs in Tianjin, with mutation and recombination occurring, yielding reassortant viruses that occasionally infect humans.

At the time of manuscript preparation, TJ/1606/18 was isolated from the fifth case of human infection with an EAS-H1N1 reported in China; the other four yielding A/Jiangsu/1/2011(H1N1) (*JS*), A/Heibei-yuhua/SWL1250/2012(H1N1) (*HB*), A/Hunan/42443/2015(H1N1) (*HN*) and A/Fujian-cangshan/SWL624/2016(H1N1) (*FJ*) [18–21]. The first case, which began in late December 2010 in a three-year-old boy in Jiangsu, resulted in death; however, the child had a history of renal disease [18,36]. The second case, which began in December 2012 in a three-year-old boy in Hebei Province, caused mild influenza-like illness [19]. The third case was a 30-month-old boy in late June 2015 in Hunan Province, who developed severe pneumonia and recovered after hospitalization treatment for 38 days. The fourth case was a 46-year-old man with severe pneumonia in October 2016 in Fujian province, and the patient died due to multi-organ failure. The fifth case reported here was a nine-year-old boy with mild ILI symptoms in December 2018, and recovered in a week without hospitalization and oseltamivir treatment. This is the first human infected with novel EAS-H1N1 in northern China, and the second case in China (the case from Fujian is the first one).

Of the four previous EAS-H1N1 virus causing human infections (Tables 3 and 4), a full-genome analysis of these viruses showed that they can be divided into two main genotypes, represented by the JS and HN viruses [33]. All eight gene segments of JS and HB belonged to EAS-H1N1 (JS-like viruses), while those of HN and FJ were derived from EAS-H1N1, A(H1N1)pdm09 and classical swine H1N1 viruses, as was the case for TJ/1606/18 (HN-like viruses). And research has indicated than HN virus showed higher infectivity, virulence, and substantially higher replication in respiratory tract of mice than JS virus [20]. TJ/1606/18 belonged to HN-like virus, but caused mild respiratory infection in human. More research about its pathogenesis in mice should be evaluated in future.

Based on previous studies, amino acid substitutions E190D and G225D/E in HA could lead to a shift in receptor-binding specificity from avian *α*-2,3-linked sialic acid (Sias) to human *α*-2,6-linked Sias, thereby increasing the binding of H1N1 virus to cells in the human upper respiratory tract [37–38]; TJ/1606/18 had 190D and 225E, as was the case in other EAS-H1N1 viruses isolated from humans (Table 6), indicating the potential risk of transmission among humans. However, the amino acid motif PSIQSR↓GL was found at the HA1/HA2 cleavage site, a characteristic of influenza viruses with low pathogenicity.

The viral polymerase is a major determinant of interspecies transmission and pathogenesis. It has been reported that L89V in PB2 [21] can enhance the polymerase activity, Q591R and E627K [21] can increase virus replication in mammals, and most recently published D701N in PB2 can indeed enhance the viral polymerase activity, viral replication, and pathogenicity in mice [17]. Here, TJ/1606/18 had L89V substitution and 627E, which exist in all five EAS-H1N1 viruses, but owned Q591R substitution and 701D, which are specific to HN-like viruses (Table 6).

Increased virus transmission between hosts has been reported for viruses carrying X99H and I368V amino acid substitutions in their PB1 polymerase [39]; TJ/1606/18 possessed 99H and 368I, which are the same as in all five EAS-H1N1 (Table 6). Furthermore, studies have shown that the influenza virus PB1-F2, encoded by a second reading frame, protein can play a key role in viral infection and virulence [24,40–41] with the protein locating in the mitochondria and leading to apoptosis [42]. However, truncated PB1-F2 with fewer than 87 amino acid residues lacks the mitochondria translocation signal and cannot function [42]. Otherwise, PB1-F2 plays critical roles in viral pathogenesis by interfering with the host immune response and influencing inflammatory responses [43]. However, PB1-F2 has dissimilar functions in different virus types/subtypes and hosts, which are determined by its length. Usually, avian influenza viruses have full-length PB1-F2 protein, enabling all functions [44], while classical swine H1N1 and A(H1N1)pdm09 viruses frequently contain truncated PB1-F2 with associated loss of function as seen in TJ/1606/18.

Recently research showed that Q357K substitution in NP protein, which is a typical human signature marker, is an adaptive signature of the influenza A viruses, allowing them to cross species barriers to circulate in humans and induces a fatal infection in mice [33]. TJ/1606/18 possessed 357K, the same as the other two HN-like viruses (HN and FJ) (Table 6), this may indicate that EAS-H1N1 already acquired the trait necessary to cause a human influenza pandemic.

Alternatively, amino acid substitutions related to increased virulence were found in PA (336M and 356R, unique in HN-like viruses), M1 and NS1 proteins of TJ/1606/18. This complex pattern of amino acid substitutions and their interaction in determining virus virulence, notably in relation to infection of humans, requires further investigation.

TJ/1606/18, like other EAS-H1N1 viruses isolated from humans, showed antiviral resistance to amantadine and rimantadine due to S31N amino acid substitution in M2 protein, but remained sensitive to neuraminidase inhibitors. Hence, at the early stage of infection by EAS-H1N1-like viruses, administration of oseltamivir or zanamivir may reduce the severity of infection [1].

In conclusion, human infection with EAS-H1N1 or a reassortant EAS-H1N1virus could result in mild or severe clinical symptoms. Whole genome sequencing has revealed a number of gene mutations encoding amino acid substitutions in a number of the virus proteins, coming together by mean of gene reassortment, which have been associated with increased virulence and likelihood of transmission to other mammals and humans. It is imperative to continue and enhance surveillance of influenza in swine as they represent a host species that can produce novel virus reasortants that could lead to another human pandemic, as seen with A(H1N1)pdm09 viruses.
